# The emergence of trophoblast cell-surface antigen 2 (TROP-2) as a novel cancer target

**DOI:** 10.18632/oncotarget.25615

**Published:** 2018-06-22

**Authors:** David M. Goldenberg, Rhona Stein, Robert M. Sharkey

**Affiliations:** ^1^ Center for Molecular Medicine and Immunology, Belleville, NJ, USA; ^2^ IBC Pharmaceuticals, Inc., Morris Plains, NJ, USA; ^3^ Immunomedics, Inc., Morris Plains, NJ, USA

**Keywords:** TROP-2, TACSTD2, sacituzumab govitecan, antibody-drug conjugates, immunotherapy

## Abstract

TROP-2 is a glycoprotein first described as a surface marker of trophoblast cells, but subsequently shown to be increased in many solid cancers, with lower expression in certain normal tissues. It regulates cancer growth, invasion and spread by several signaling pathways, and has a role in stem cell biology and other diseases. This review summarizes TROP-2's properties, especially in cancer, and particularly its role as a target for antibody-drug conjugates (ADC) or immunotherapy. When the irinotecan metabolite, SN-38, is conjugated to a humanized anti-TROP-2 antibody (sacituzumab govitecan), it shows potent broad anticancer activity in human cancer xenografts and in patients with advanced triple-negative breast, non-small cell and small-cell lung, as well as urothelial cancers.

## INTRODUCTION

The challenge of cancer treatment remains selectivity - attacking the cancer while minimizing collateral damage to normal cells. Since cancer cells are altered in some fundamental way, it is logical to think that these differences are manifested by genetic changes and/or the expression of new protein molecules. If accessible, these can serve as therapeutic targets providing tumor specificity. Indeed, these considerations led to the era of targeted cancer therapies, which include agents that block the growth and spread of cancer by interfering with specific molecules critical to the features of malignancy, such as proliferation, progression, and spread; hence, the development of molecularly-targeted drugs, more generally comprising what is termed precision medicine.

Precision medicine now represents the focus of most anticancer therapies in development, based on the identification of targetable gene mutations and marker proteins that lead to more selective methods of prevention, diagnosis and therapy [1]. For example, the BCR-ABL fusion protein made from two different genes was found to promote the proliferation of leukemic cells, thus proving to be a useful therapeutic target (imatinib mesylate) [2, 3]. However, even before the era of precision medicine, many proteins produced in elevated quantities by tumor cells, such as Bence-Jones protein, beta human chorionic gonadotrophin (β-HCG), alpha-fetoprotein (AFP), carcinoembryonic antigen (CEA), prostatic acid phosphatase, and human epidermal growth factor receptor (EGFR), and EGFR-2 (HER2), were used as circulating biomarkers of disease activity aiding in immunodiagnosis [4] or even as targets for radionuclide- or drug-conjugated antibodies [5, 6].

This review focuses on TROP-2, which is increased in a large variety of solid cancer cells and appears, both as a gene and protein, to affect signaling pathways involved in cancer proliferation, migration, invasion, and metastasis. Its role as a biomarker for cancer therapeutics also is addressed.

## DISCOVERY OF TROP-2

Although first described almost 40 years ago as a cell surface marker of trophoblast cells [7], TROP-2 (trophoblast cell-surface antigen 2) was rediscovered in ensuing years as tumor-associated calcium signal transducer 2 (TACSTD2), membrane component chromosome 1 surface marker 1 (M1S1), gastrointestinal antigen 733-1 (GA733-1), and epithelial glycoprotein-1 (EGP-1) [8, 9].

The expression, role, and function of TROP-2 became of interest to us in about 1990, when we developed a monoclonal antibody that reacted with a glycoprotein expressed by many different cancer types. In our initial reports we referred to this as EGP-1 [10–13]. The recognition that this antibody recognized a unique marker of trophoblast and neoplastic cells, [renamed TROP-2 once it was identified as the same antigen called by different designations [10–12]], was fortuitous, because it was developed in the search of a marker of non-small-cell lung cancer (NSCLC) [10, 11]. The murine monoclonal antibody, designated RS7-3G11 (later shortened to RS7), was developed by immunizing mice with a cell membrane preparation isolated from a surgical specimen of a squamous NSCLC. RS7 bound strongly to lung, breast, and prostate cancer cell lines, weakly to colon cancer cell lines, and was absent in a lung fibroblast cell line, as well as granulocytes, monocytes and lymphocytes. Immunohistology of fresh frozen tissues showed that RS7 bound to breast, colon, renal, lung, and prostate cancers (33/40 [83%] positive, with 22/33 [67%] staining strongly positive). Weak staining also was observed in 16/20 normal tissues from the breast, colon, kidney, liver, lung, and prostate [10, 11].

The molecular properties of the antigen bound by RS7 were identified in 1993 (11), when the EGP-1 antigen was described as a 46-kDa glycoprotein (35 kDa when deglycosylated) [12–14] that was phosphorylated by protein kinase C (PKC), this occurring specifically on serine 303 in the cytoplasmic domain [14]. This suggested that TROP-2 has a role in signal transduction across the cell membrane. Particularly interesting was the observation that 50% the RS7 antibody bound to the cell surface internalized within ~1 h [11, 15].

Early studies recognized that TROP-2 is involved in regulating cancer growth and invasion [7, 16, 17]. The gene, *TACSTD2*, was mapped on chromosome 1p32 [18]. The 36-kDa nascent polypeptide, which is post-translationally modified by N-linked glycosylation, forms a type-1 transmembrane protein that is distinct from its sister molecule, epithelial cell adhesion molecule (EpCAM or EGP-2) [11–13].

## TROP-2 PROPERTIES AND FUNCTIONS

**Figure 1:**
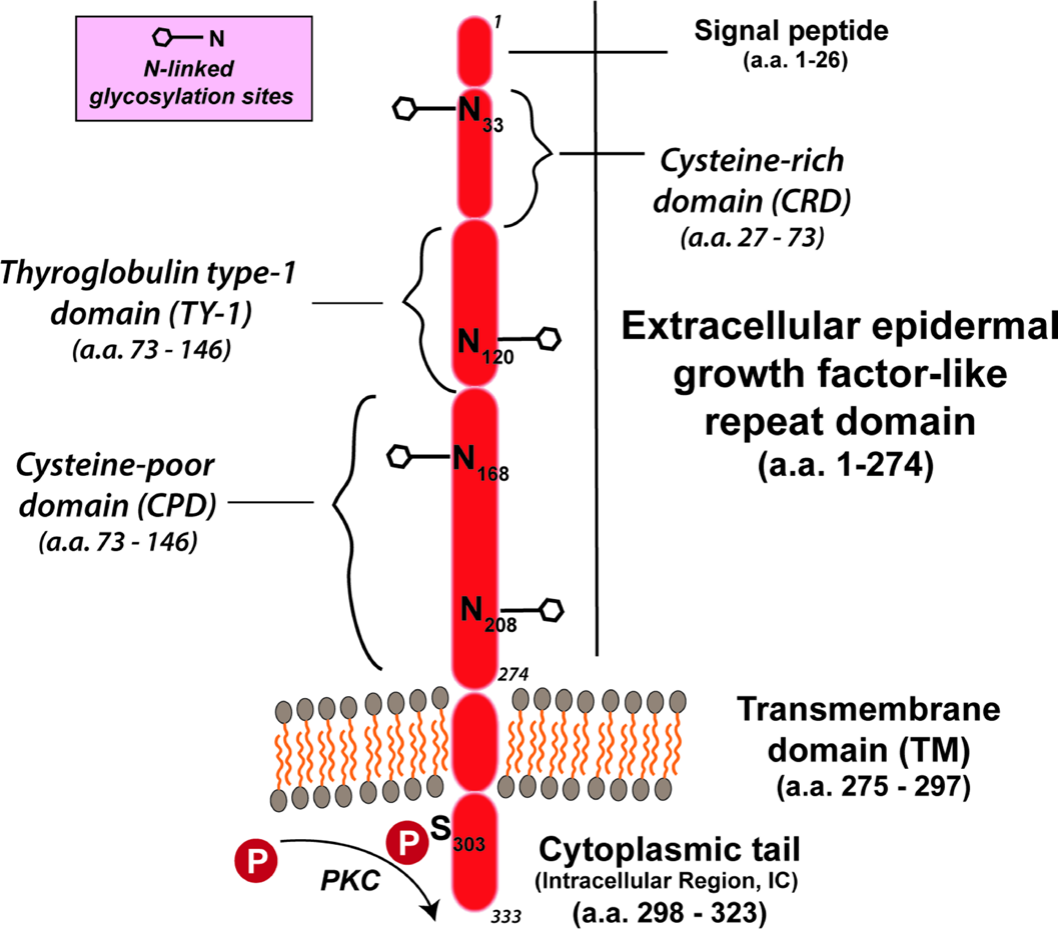
TROP-2 structure [as per Vidmar *et al*. [36]] TROP-2 contains a 274-amino-acid extracellular epidermal growth factor-like repeat portion that contains 3 domains, a cysteine-rich domain, a thyroglobulin type-1 domain, and a cysteine-poor domain. The molecule traverses the membrane and terminates with a cytoplasmic tail that has a serine at position 303 that can be phosphorylated. The molecule has 4 N-glycosylation sites in the extracellular domain.

The *TROP-2/TACSTD2* gene has been sequenced in several mammalian species [19–21]. Its intronless gene encodes a 35–46-kDa protein having 323 amino acids, comprised of a large extracellular domain, a single transmembrane domain, and a short intracellular, or cytoplasmic tail (Figure 1) [14, 16, 22]. It encodes a transmembrane Ca^++^-signal transducer [14, 23]. The single transmembrane region of TROP-2 has 23 amino acids and a 26-amino acid cytoplasmic region (Figure 1) [22]. The cytoplasmic tail shows structural and sequence homology to a HIKE domain [24–26] and, as described, contains a serine residue (S303) that is phosphorylated by PKC [14], as well as a phosphatidyl-inositol 4,5-bisphosphate (PIP_2_) binding site [27]. The signaling peptide of TROP-2 protein in the cytoplasmic tail is composed of 30 amino acids; the extracellular domain has 244 amino acids, with 12 cysteine residues [22]. There is a thyroglobulin type 1 repeat domain [19, 28], and upstream from this is an epidermal growth factor-like domain [19, 28].

**Figure 2:**
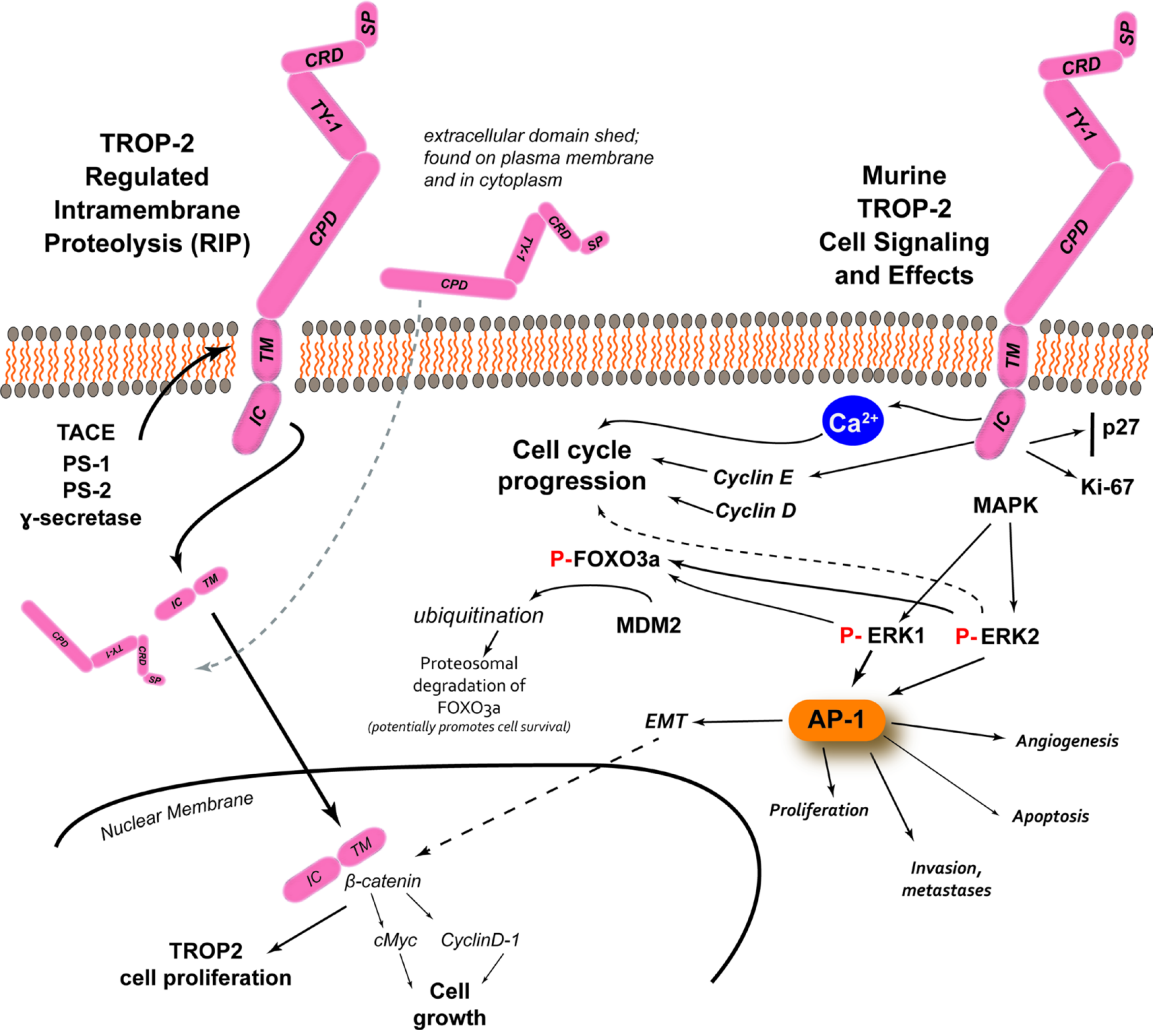
TROP-2 processing, cell signaling and its effects [adapted from Shvartsur and Bonavida, [9]] In prostate cancer, studies have found several enzymes involved in the cleavage of the TM-IC portion of the molecule, with the extracellular domain remaining associated with the plasma membrane or found in the cytoplasm. β-catenin colocalized with TM-IC in the nucleus, which can lead to TROP-2-driven proliferation, but also it can upregulate cyclin D1 and c-myc, which can lead to cell growth. Apart from its processing, TROP-2 has the potential to influence several intracellular signaling pathways that can then lead to several different events. The phosphorylation of serine-303 appears be involved in the release intracellular Ca^2+^, which can activate Raf and NF-κB pathways, and stimulate MAPK signaling and cell cycle progression. TROP-2 can increase cyclin D1 and cyclin E, which together with ERK1/2 can mediate cell cycle progression. Studies with murine TROP-2 have revealed the stimulation of MAPK and downstream upregulation of phosphorylated ERK1/2 can induce the AP-1 transcription factor that can regulate a number of tumor-associated target genes involved in angiogenesis (e.g., via VEGF), proliferation (e.g., via cyclins and CDKs), apoptosis (e.g., via BCL-2, FasL), and invasion and metastasis (e.g., via matrix metalloproteinases, podoplanin, Ezrin, and CD33), as well as epithelial to mesenchymal transition (EMT) that can interact with β-catenin to affect cell growth. Studies with murine TROP-2 also found that increased ERK activity can induce the phosphorylation of FOXO3a, followed by its ubiquitination by mouse double minute 2 (MDM2), with its subsequent degradation. The degradation of FOXO3a can promote cancer cell survival.

TROP-2 can affect signaling by insulin-like growth factor-1 (IGF-1) [29], and by interaction with neuregulin 1, inhibits ErbB3 (HER3) in head and neck squamous cell cancer [30]. Having a HIKE domain and a PIP_2_ binding site, as well as the serine phosphorylated by PKC, indicate that TROP-2 is involved in calcium signaling. This Ca^2+^ release is thought to induce mitogen-activated protein kinase (MAPK) signaling and advance the cell cycle [31]. The phosphorylation of TROP-2 by increased PKC could in turn activate the Raf and NF-κB pathways [8] (Figure 2).

The *TACSTD* gene family also contains the gene that codes for *Trop-1*, also known as gastrointestinal antigen 2 (*GA733-2*), *EGP-2* (12), or *EpCAM*. Although *EpCAM* is coded by a different gene on chromosome 2p21, it shares 49% sequence identity and 67% sequence similarity with *TROP-2*; EpCAM has 314 amino acids and is a 35 kDa protein [19, 32]. They are both type I transmembrane proteins, but unlike *TROP-2* that is intronless, *EpCAM* has nine coding exons; 1–6 code for the extracellular domain, while exon 7 is the transmembrane domain and exons 8–9 code for the intracellular region [33].

TROP-2 and EpCAM have similar cysteine positions and distributions of hydrophilic and hydrophobic residues, but EpCAM only has three N-glycosylation sites in contrast to four for TROP-2 [34]. There are also differences in the intracellular tails that account for different intracellular signaling and thus different functions and distributions between TROP-2 and EpCAM. The highest homology is in the thyroglobulin repeat and the single transmembrane domains [19, 20]. The promotor regions of *EpCAM* and *TROP-2* are unrelated, resulting in different expression patterns [35].

TROP-2 has been reported to bind to several proteins, such as IGF-1, claudin-1 and -7, cyclin D1, and PKC (Figure 2). At least IGF-1 could be a ligand for TROP-2, modulating IGF-1 signaling and activating PIP_2_ and Ca^2+^. TROP-2 may also complex with IGF-1, presumably blocking IGF-1 signaling [29]. In contrast to the experience with most cancers, high expression of TROP-2 suppresses lung cancer growth by attenuating IGF-1R signaling, probably by complexing with IGF-1 [36]. Claudin-1 and -7 are transmembrane proteins that bind to TROP-2's ectodomain, which may affect maintaining tight junctions at the epithelial surface, possibly preventing claudin degradation [36].

TROP-2 also activates the ERK1/2 (extracellular signal regulated kinase)-MAPK pathways, contributing to cell progression [29], and could play a role in deregulating stem cell functions via Notch, Hedgehog and Wnt pathways (Figure 2) [31]. As mentioned, the MAPK pathway is also stimulated when Ca^2+^ is increased. Thus, TROP-2 generally increases the levels of phosphorylated MAPK, affecting cell cycle progression. Further, activation of ERK has been reported in several tumor types that overexpress TROP-2, and this ERK1/2 activation is thought to promote tumor survival by having anti-apoptotic effects [31].

## TROP-2 IN CANCER

TROP-2 has been studied in embryonic and fetal development [20], but most studies have focused on its role in cancer. As mentioned, with rare exceptions, it has been linked to increased tumor growth and enhanced proliferation, cell migration and anchorage-independent growth, and is overexpressed in most human solid epithelial cancers, such as oral, head-and-neck, thyroid, lung, esophageal, gastric, colorectal, pancreatic, breast, renal, uterine, cervical, ovarian cancers, and glioma [37–50]. These authors proposed that TROP-2 is a prognostic marker in most of these cancers (reviewed in [38]). The ectopic production of TROP-2 in cancer cells in culture has been shown to transform murine fibroblasts when injected into mice, suggesting at first that *TROP-2* is an oncogene [51]. Further studies modified this view [52], so that although the level of TROP-2 generally influences malignancy, it may not by itself be a true oncogene. Nevertheless, knockdown of the *TROP-2* gene by small-interfering (si) RNA in colon, breast, cervical, lung, and ovarian cancer cells inhibits their proliferation, invasion, and the formation of colonies *in vitro* [43, 51, 53–55]. The knockdown of TROP-2 in gallbladder cancer reduces cell proliferation, invasion and migration, but also inhibits vimentin and increases E-cadherin expression linked to epithelial-mesenchymal transition (EMT) [56].

Tumor growth in mice is related to levels of *TROP-2* mRNA, where high expression of TROP-2 is found in the largest tumors [53]. Inhibition of TROP-2 with anti-TROP-2 antibodies decreases migration of colon and breast cancer cells *in vitro* [51, 57], while overexpression increases the migration of pancreatic cancer cells [31]. High TROP-2 expression is also correlated with increased metastasis in patients with different cancer types (oral squamous, thyroid, some esophageal, gastric, colorectal, pancreatic, ovarian, uterine, cervical, prostate, and urinary bladder), but is not upregulated in others (e.g., head and neck and certain lung cancers, such as lung adenosquamous and squamous cell carcinoma histology) (reviewed by [9, 37, 38, 58]). In gastric cancer, there appears to be a relationship between increased TROP-2 and amphiregulin coexpression and poor survival [59]. In HNSCC, TROP-2's gene (*TACSTD2*) has been identified as the target of microRNA, miR-125-1, resulting in dysfunction of the MAPK pathway [60]. The effects of TNF-α on regulation of TROP-2 expression in colon cancer indicated that low concentrations increase TROP-2 protein expression, while higher concentrations of TNF-α have a decreasing influence [61]. These authors also related TNF-α stimulation to the ERK1/2 pathway, because a specific inhibitor of ERK1/2 suppresses the cytokine's upregulation of TROP-2 [61]. However, the role of TROP-2 in hematological tumors is unclear, being expressed in Hodgkin lymphoma and chronic lymphocytic leukemia [9], but not in anaplastic large cell lymphoma [62].

From a functional perspective, it is important that *TROP-2* fuses with *cyclin D1* (bicistronic *cyclin D1-TROP-2*) to become an oncogene [63]. This binding of the two mRNA molecules affects the stability of *cyclin D1*, and as a chimera, can increase cell longevity and proliferation [8], as well as transformation. Indeed, tumor growth can be inhibited by silencing this fusion protein [29]. In invasive ductal breast cancer, elevated expression of *TACSTD2* and *cyclin D1* at the mRNA and protein levels were shown to be independent prognosticators of a poor outcome [42].

Although both TROP-2 and EpCAM predict a poor prognosis when overexpressed in breast and ovarian cancers, in small-sized adenocarcinoma they have opposite biological effects, with TROP-2 having a negative prognostic effect while EpCAM indicating a favorable prognosis [64]. From an evolutionary perspective, *EpCAM* is believed to have given rise to *TROP-2* by retroposition prior to the divergence of avian and mammalian lineages [19].

Summarizing, the increased expression of TROP-2 is reported to be “necessary and sufficient” for stimulation of cancer growth, while a bicistronic cyclin D1/TROP-2 mRNA chimera is an oncogene [53, 65]. Importantly, elevated expression is associated with more aggressive disease and a poor prognosis in several cancer types [8, 9, 20, 37–50, 66]. This elevated tumor expression of TROP-2 does not appear to circulate in the blood, yet there is a report that some esophageal cancer patients have circulating antibody to this biomarker [67]. There are at least six major signaling pathways involving TROP-2 in cell proliferation, but its precise role in these and which pathway(s) are critical in different cancers and in different therapeutic approaches remain to be elucidated.

The TROP-2 signaling network involved in cancer proliferation has been studied extensively by Alberti and coworkers [23, 27, 41, 53], and reviewed recently by Shvartsur and Bonavida [9]. These authors have also reviewed the *TACSTD2* transcription control network, indicating that the TROP-2 gene is connected with many transcription factors. Importantly, TROP-2 upregulation activates CREB1 (cyclic AMP-responsive-element binding protein), Jun, NFκB, Rb, STAT1 and STAT3 via induction of the cyclin D1 and ERK/MEK pathways [41].

## TROP-2 IN STEM CELL BIOLOGY AND OTHER DISEASES

TROP-2 has also been found in stem cells of various tissues, particularly in basal cells. For example, in murine and human prostate, the basal cells expressing TROP-2 have self-renewal, regeneration and differentiation properties [68, 69]. After liver injury, undifferentiated oval cells express TROP-2 [70]. Endometrial-regenerating cells also express TROP-2 [71]. These findings support the view that TROP-2 may be implicated in the regulation of stem cell growth and regeneration in several tissues, perhaps playing a role in increased cell proliferation, such as hyperplasia.

Studies in the prostate reveal a potential role of TROP-2 in oncogenesis. The normal prostate is composed of three primary cell types: luminal, basal, and neuroendocrine. The luminal cells express the secretory proteins, such as prostate-specific antigen (PSA) and cytokeratin-8, and high levels of androgen receptor, while the basal cells are below and express other markers and less androgen receptor. The luminal cells have been considered to be precursors of adenocarcinoma, but Goldstein *et al*. showed that basal cells expressing TROP-2 can form prostate carcinoma in immunodeficient mice [69]. Thus, basal cells expressing TROP-2 and CD44 can transform to tumors of the luminal phenotype [68, 69, 72, 73]. This is consistent with studies implicating TROP-2 as a key regulator of β1 integrin activities and promoting prostate cancer cell motility [74, 75]. Interestingly, TROP-2^+^ exosomes purified from prostate cancer promote migration of TROP-2-negative prostate cancer cells on fibronectin, suggesting that TROP-2 could induce cells lacking TROP-2 to gain TROP-2 regulatory properties affecting migration [47].

TROP-2 also has been implicated in stem cell changes in cardiomyopathy and pulmonary disease. It was demonstrated in mice that although c-kit^+^/TROP-2^+^cells are rarely expressed by normal myocardium, their frequency increases significantly following the induction of a myocardial infarct [76]. Evidently these putative stem cells participate in proliferation and survival following this wound, leading to activation of the MAPK cascade via TROP-2-induced signal transduction, similar to oncogenesis. Thus, activation of TROP-2 could provide protection to the damaged myocardium by promoting proliferation of c-kit^+^ stem cells.

It has also been reported that increased expression of TROP-2 in airway basal cells could contribute to proliferation of such cells derived from smokers with chronic obstructive pulmonary disease (COPD) [77]. TROP-2 basal cells exhibit improved proliferation with activation of the ERK1/2 phosphorylation signaling pathway, and with an EMT-like change, not unlike the effects found in oncology. Down-regulation of TROP-2 by siRNA significantly reduces the proliferation of basal cells from these COPD patients, mitigating the EMT-like features. These changes may also be implicated in the genesis of squamous cancer in smokers, where TROP-2 is also a targetable therapeutic marker.

## *TACSTD2* MUTATION CAUSES GELATINOUS DROP-LIKE CORNEAL DYSTROPHY (GDLD)

In 1999, Tsujikawa *et al*. [78] identified an inherited mutation in the TROP-2 gene, *TACSTD2*, as the cause of GDLD, which was first recognized as an autosomal recessive disease leading to the development of severe corneal amyloidosis and blindness in Japanese in 1914 by Nakaizumi [79], but later also identified in other nationalities [80], including other mutations of *TACSTD2* [80]. The most frequent mutation [82.5% of all mutations of this gene in Japanese] [78] involves glutamine being replaced with a stop codon, Q118X, at codon 118, in the thyroglobulin repeat domain. The result is a truncated protein lacking the transmembrane domain, with a decrease or absence of certain tight junction proteins, including claudin 1, 4, and 7 [81, 82]. This results in lactoferrin penetrating the corneal epithelium and the onset of amyloidosis, causing blindness [82]. This role of TROP-2 in barrier function and tight junction is likely also related to its effects on the adhesion and migration of cancer cells. However, no mutations in *TACSTD2* have been reported in cancer.

## CANCER THERAPIES TARGETING TROP-2

### Radioimmunotherapy

Radioiodinated RS7 targets human cancer cell line xenografts selectively and specifically [10, 15, 83, 84], but its internalization properties influenced the selection of radionuclide; i.e., residualizing radioiodine or radiometals (e.g., ^111^In/^90^Y) increase tumor accretion and improve efficacy [83, 85–89].

Interestingly, despite internalization, TROP-2 is an appropriate target for a two-step pretargeting approach, using a bispecific antibody with one arm targeting TROP-2 and another arm binding a hapten of 3 amino acids [90–93]. Since a pretargeting method relies on the retention of the bispecific antibody on the cell surface, there was concern that an internalizing anti-TROP-2 antibody would not be an ideal first targeting agent because of a short residence time on the tumor cell surface. However, even after a delay of about one day, *in vitro* studies showed that a sufficient amount of the bispecific antibody remains on the cell surface to permit subsequent targeting by the radiolabeled hapten [90]. Hence, the 2-step pretargeting approach with TROP-2 can provide excellent imaging and therapeutic results using a radiolabeled hapten [84, 90–92].

### Other TROP-2-targeted therapeutics

Unlike radionuclides that can exert a therapeutic effect without requiring internalization because of a crossfire effect, other cytotoxic agents, such as drugs and toxins, require internalization and processing within the cell to exert the desired effects. Thus, the internalization properties of the RS7 antibody targeting TROP-2 provide opportunities for the delivery of cytotoxic compounds to cancers expressing TROP-2. For example, Chang *et al*. [94] first reported the development of a recombinant fusion protein between the humanized version of the RS7 antibody (hRS7), where a deglycosylated mutant form of the RNase toxin, Rap (*Rana pipiens*) was fused to the N-terminus (i.e., on the V_L_) of the light chains, giving a substitution ratio of 2 Rap/IgG. Liu *et al*. [95] subsequently reported the development of a unique set of fusion proteins, utilizing the Dock-and-Lock^®^ (DNL^®^) procedure [96–98] that paired the targeting of TROP-2 using the hRS7 IgG with the cytotoxic activity of Rap. Two agents were prepared, one with 4 Rap molecules attached to the C_H_3 heavy chain of the IgG and the other attaching 4 Rap molecules to the C_K_ light chain. These constructs were tested primarily in triple-negative breast cancer (TNBC) cell lines, where they showed higher potency than the previously-described recombinant construct [94], with EC_50_ values in the subnanomolar level against several cancer cell lines [95]. In xenograft models, superior therapeutic responses also were reported [94].

## ANTIBODY-DRUG CONJUGATES (ADCS)

This section focuses on two anti-TROP-2 ADCs that have been studied clinically, sacituzumab govitecan, using the topoisomerase inhibitor, SN-38 (an irinotecan metabolite) [66, 99–107], and another agent, RN927C, coupled to a derivative of the microtubule inhibitor, auristatin [108, 109]. Other TROP-2-targeted therapeutics have been described preclinically, one using a nanoparticle (carboxymethyl dextran) carrier linked with doxorubicin, which has activity in the MDA-MB-231 breast cancer cell line representative of TNBC [110], and another, utilizing doxorubicin conjugated to an anti-TROP-2 Fab, with activity *in vitro* and *in vivo* against pancreatic cancer [111].

### Sacituzumab govitecan (IMMU-132)

**Figure 3:**
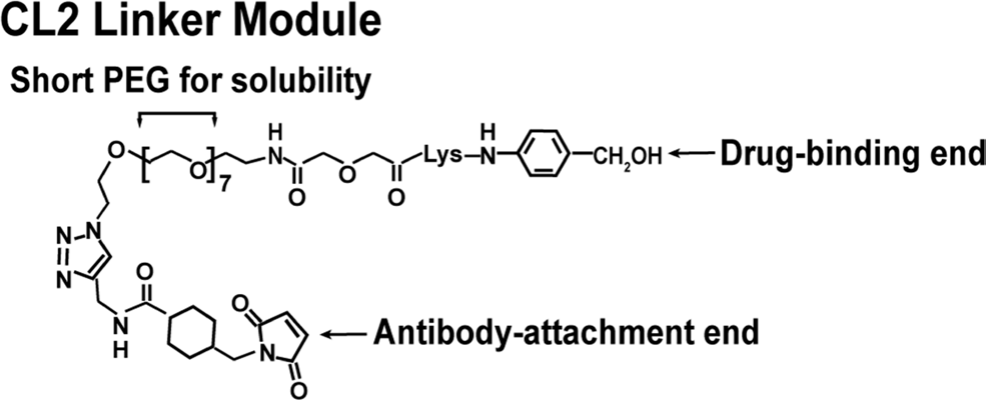
Representation of structure of the CL2A linker used to bind SN-38 to the anti-TROP-2 IgG to form sacituzumab govitecan The CL2A linker forms a water-soluble SN-38 conjugate with excellent yields. Water solubility is achieved by inserting a short polyethylene glycol segment. The linker binds to the 20th position of SN-38, forming a pH-sensitive carbonate bond. Binding to the 20th position stabilizes the lactone ring. A maleimide at the end of the linker will enable a stable thioether bond with sulfhydryl moieties formed after mild reduction of the antibody. This process will bind up to 8 moieties to the antibody without affecting antigen binding.

The significant challenge for any ADC is the chemistry linking the drug to the antibody. Moon *et al*. [112] focused on evaluations of six linker-SN-38 derivatives that were bound through various modifications of the 20th position of SN-38's lactone ring, using as many as 5 antibodies with specificities for antigens found in hematopoietic and solid tumors. After consideration of challenges in chemistry, solubility, yields, retention of SN-38 potency and antibody binding, stability of the conjugate in buffer and serum, and, finally, activity when administered to nude mice bearing appropriate human cancer xenografts, cross-linked (CL) derivatives, designated CL2-SN-38 and CL2-SN-38(Et), were studied. In each case, 5-7 SN-38 moieties were coupled to the antibodies without compromising antibody binding to their respective antigen. After extensive *in vitro* and *in vivo* testing, the CL2 linker was selected (Figure 3), but with a further modification that eliminated the cathepsin-B cleavable Phe-Lys peptide, making the derivative known as CL2A the preferred linker for clinical use [99].

The next question was whether the therapeutic benefits of this type of conjugate are driven by the selectivity of the antibody or perhaps merely a slow release of the SN-38. In non-clinical studies, sacituzumab govitecan was tested against human epithelial cancer xenograft models of various gastrointestinal cancers, including gastric and pancreatic cancers, lung cancers, non-TNBC and TNBC, prostate, and cervical cancers [66, 99, 100]. Comparisons included SN-38, irinotecan (IRI), or a non-targeting ADC, with sacituzumab govitecan providing improved therapeutic outcomes in nearly every model studied. However, *in vitro* evidence of therapeutic selectivity based on the antibody binding was lacking, because the cytotoxicity assay used most commonly relied on an extended exposure (e.g., for 4 days) to the drug [66, 99, 100]. Since the SN-38 is released from the conjugate during this incubation period, specificity could not be demonstrated. Ultimately, an assay monitoring the formation of double-stranded DNA breaks showed definitively that a conjugate with the TROP-2 antibody enhanced DNA damage [66].

As mentioned above, *in vivo* testing illustrated a selective improvement in therapeutic responses attributed to the TROP-2 conjugate, but with the notable exception of the SK-MES-1 lung cancer cell line (derived from squamous cell carcinoma), where the therapeutic effects of sacituzumab govitecan are not significantly different from IRI. We reported [99] that TROP-2 expression, as determined by flow cytometry median fluorescence intensity, is lower in these cells than most of the other cancer lines tested. However, this cell line is also less sensitive to SN-38. Thus, while it is logical to expect efficacy to be driven by the expression level of a given antigen, there are other factors to consider, such as a tumor's sensitivity to the drug being used, and other physiological issues that might affect the delivery of the conjugate/drug to tumors *in vivo*.

In an effort to isolate how antigen expression might impact the *in vivo* efficacy of sacituzumab govitecan, cDNA of human TROP-2 was transfected into the MDA-MB-231 TNBC cell line, which only expresses ~32,000 copies of membrane-bound TROP-2 per cell, in order to develop clones with increased expression [113]. Unlike SK-MES-1, where sacituzumab govitecan and IRI have a similar therapeutic benefit, MDA-MB-231 is unresponsive to either agent [113], and therefore it was of further interest to determine whether enhancement of TROP-2 expression yields improved selective sensitivity to sacituzumab govitecan. The results clearly indicate that (*i*) transfection of the MDA-MB-231 with human TROP-2 cDNA does not appreciable alter its sensitivity to IRI, and (*ii*) increasing TROP-2 expression ~4 fold (from ~30,000 to 120,000) significantly enhances sacituzumab govitecan's therapeutic activity. This enhancement is clearly driven by the TROP-2 specificity of the ADC, since responses to a non-targeting ADC are not substantially different between the parental and transfected cells.

A final important consideration for an ADC is minimizing collateral damage to normal tissues, whether a consequence of the release of the drug or a result of its selective targeting to the antigen on normal cells (on-target non-specificity). Given TROP-2's expression in a number of normal tissues, a study was performed in Cynomolgus monkeys who express TROP-2 in similar tissues as humans. At the highest dose of sacituzumab govitecan tested, the monkeys experienced severe neutropenia and diarrhea, typical symptoms for SN-38/IRI toxicity. However, histological assessment of the tissues revealed most TROP-2-expressing tissues had minimal toxicity, with recovery by the end of study [99]. Thus, it appeared that there was SN-38-directed toxicity, but not selective toxicity based on TROP-2 targeting, with many of the TROP-2 expressing normal tissues having minimal damage. An assessment of the clearance properties of sacituzumab govitecan in the monkeys revealed that the agent released the SN-38 payload at a similar rate as predicted from *in vitro* serum stability studies, while the IgG had a more protracted clearance.

Another line of nonclinical investigation was undertaken to confirm the improved delivery of SN-38 via the anti-TROP-2 antibody, compared to IRI. Sharkey *et al*. [114], using two human tumor xenografts, reported that the concentrations of SN-38 in tumor xenografts showed a 20- to 136-fold improvement for the ADC *vs*. IRI. This clearly demonstrated the advantage of using the TROP-2-targeting antibody to deliver the topoisomerase-1-inhibiting drug. They also reported that levels of glucuronidated SN-38 (SN-38G) in the animals' serum were much lower with the ADC. SN-38G is a detoxified derivative of SN-38; however, it recycles via the enterohepatic pathway, where it can then be converted to SN-38 by bacterial enzymes in the intestine, leading to late diarrhea in IRI therapy. The lower levels of SN-38G found with the ADC suggested severe diarrhea may be reduced. Thus, these results provided the impetus to pursue clinical studies with sacituzumab govitecan.

### Clinical trials with sacituzumab govitecan

**Figure 4:**
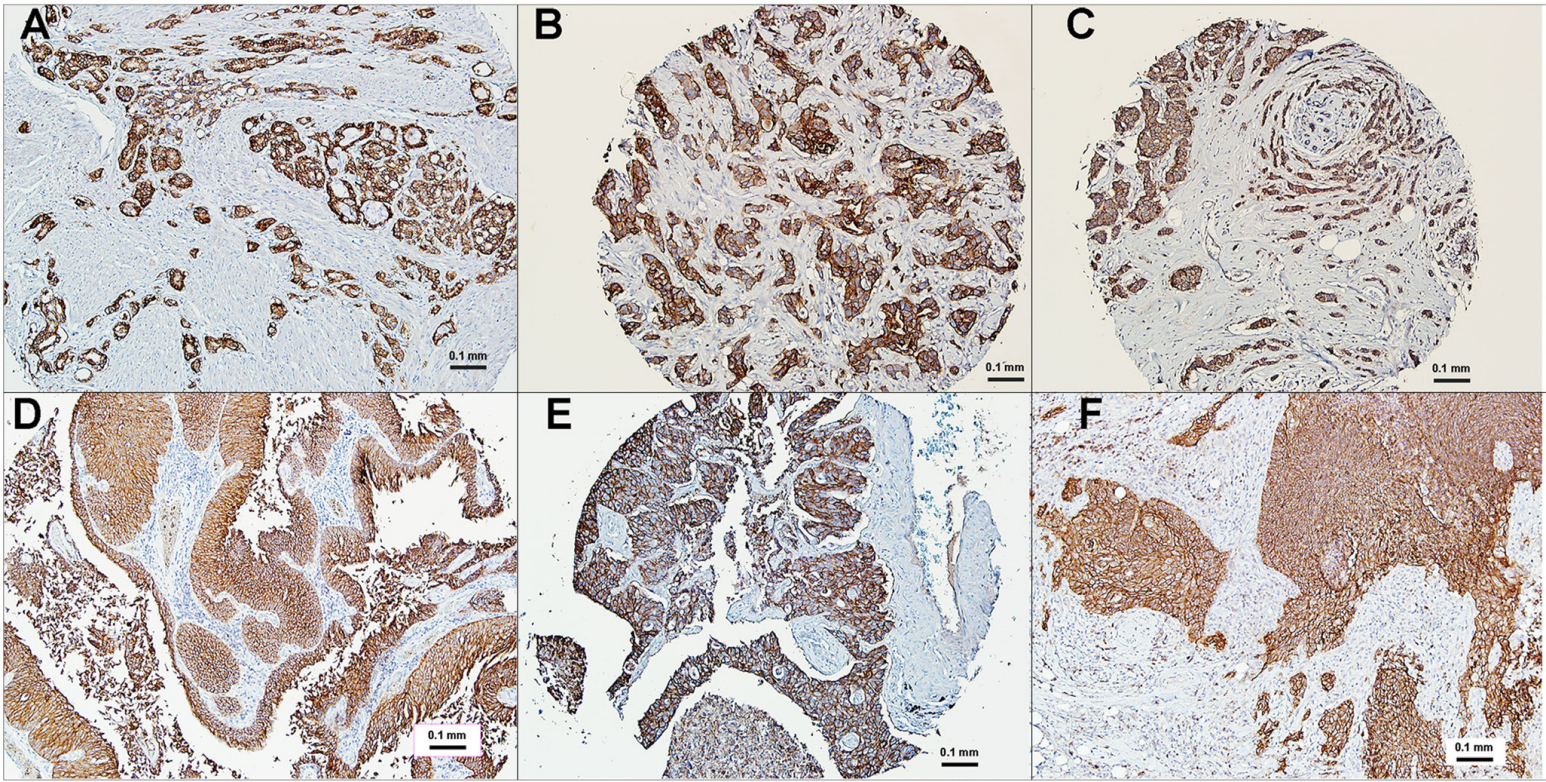
Immunohistochemical localization of TROP-2 in human cancers Polyclonal antibody to human TROP-2 was used to reveal TROP-2 localization in tumor sections, mostly from commercial microarrays (bar = 0.1 mm). (**A**) prostate cancer, (**B**) triple-negative breast cancer, (**C**) estrogen-receptor and HER2 positive breast cancer, (**D**) urinary bladder cancer, (**E**) non-small-cell lung cancer, (**F**) small-cell lung cancer. All specimens selected based on their moderate (2+) to strong (3+) expression of TROP-2. Staining is found both on the membrane and in the cytoplasm.

The Phase I clinical study with sacituzumab govitecan was designed as a basket trial including patients with diverse metastatic epithelial cancers who had failed conventional treatments, with 10 indications studied initially (i.e., colorectal [CRC], gastric, hepatocellular, non-small-cell [NSCLC] and small-cell lung [SCLC], ovarian, pancreatic, prostate, TNBC, and urothelial cancers [UC]) [101]. Prescreening for TROP-2 expression was not required, since immunohistology studies using tissue microarrays of these cancers indicated a higher than 80% positivity, with most having moderate to strong staining (Figure 4). As mentioned, TROP-2 is reported as a prognostic marker for most solid cancers (reviewed in [38]). At present, however, whether there is a difference in expression between primary and metastatic tumors has not been determined.

Patients were intended to receive multiple treatment cycles of sacituzumab govitecan, starting at a dose of 8 mg/kg given on days 1 and 8 of a 21-day treatment cycle. Treatment continued until progression or when no longer tolerated. Dose delays of up to 2 weeks and dose reductions (e.g., by 25%) were permitted. Responses were assessed every 8 weeks, with a confirmatory study performed within 4–6 weeks of any response, complying with RECIST 1.1 criteria for objective responses (114).

Gastrointestinal cancers represented the largest enrollment of the 25 patients included in this Phase I assessment, with >30% tumor shrinkage found in 3 cases (SCLC, TNBC, and CRC). The maximum tolerated dose (MTD), based on the tolerance to the first treatment cycle, was determined to be 12 mg/kg, with neutropenia as the dose-limiting toxicity. However, since the treatment was intended to be given over multiple cycles, and because it appeared that multiple cycles were tolerated better at the starting dose levels of 8 and 10 mg/kg [101], the Phase II portion of this basket trial was expanded first to include enrollment at each of these two dose levels.

Ocean *et al*. [115] reported an overview of the data from patients enrolled with diverse cancers, with 81 patients first given a starting dose of 8 mg/kg, followed by enrollment of 97 given 10 mg/kg. The study focused on evaluating safety and pharmacokinetics, with an initial examination of efficacy, based on overall response rate and clinical benefit rate, in 4 cancer indications having enrollment of more than 20 patients. The pharmacokinetics of the agent and its products (SN-38 and SN-38G) was similar at the 2 dose levels. As found in animal studies, levels of SN-38G in the serum were markedly less than that reported for IRI therapy [116, 117]. An examination of safety, including the ability to tolerate sequential treatments at the starting dose level, showed the 8 mg/kg dose level was tolerated somewhat better, but the tolerance of 10 mg/kg dose level within the first cycle of treatment was acceptable. Dose delays or reductions were almost always based on neutropenia, which occurred at a similar rate as reported for IRI therapy, while cases of grade >3 diarrhea was only 10% in the 10 mg/kg group, which was much lower than for IRI therapy. We speculated that lower concentrations of SN-38G in the serum might explain this difference, since the re-circulation of SN-38G is suspected to be responsible for late diarrhea in IRI therapy [117, 118]. A review of the initial data for therapeutic response in patients with CRC, NSCLC, SCLC, and TNBC indicated a trend for improved response rates and clinical benefit in patients given the starting dose of 10 mg/kg. Thus, with an acceptable safety profile in a diverse population of cancer patients, and with initial evidence of improved responses, 10 mg/kg was selected as the starting dose for sacituzumab govitecan monotherapy.

The expanded Phase II clinical experience with sacituzumab govitecan in patients given 10 mg/kg as their starting dose has been reported for TNBC, NSCLC, SCLC, and UC [102–107]. In all these indications, the safety and pharmacokinetics have been similar to that reported for the Phase I and the initial evaluation of the expanded Phase II trials [101, 115]. The majority of these cases express moderate to high levels of TROP-2 in their archived tumor specimens, currently making selection based on TROP-2 expression unnecessary. The initial response-related results from the phase II trials in TNBC; metastatic hormone-positive, HER2-negative breast cancer; NSCLC; SCLC; and UC are summarized in Table 1.

**Table 1:** Summary of published results on phase II trials with sacituzumab govitecan

Cancer type^1^ [Ref]	Number of patients	Confirmed % ORR^2^	Median DoR (months)	Median PFS (months)^3^	Median OS (months)^3^
TNBC [106]	69	30	8.9	6.0	16.6
HR^+^/HER2^−^ BC [135]	54	31	NR	NR	NR
NSCLC [104]	54	19	6.0	5.2	9.5
SCLC [103]	50	14	5.7	3.7	7.5
UC [105]	41	34	12.9	7.2	15.5

^1^TNBC, triple-negative breast cancer; HR^+^/HER2^−^ BC, hormone-receptor positive, HER2-negative metastatic breast cancer; NSCLC, non-small cell lung cancer; SCLC, small-cell lung cancer; UC, urothelial cancers.

^2^% ORR, objective response rate = (complete response + partial response)/number of patients.

^3^PFS, progression-free survival; OS, overall survival. Based on the number of intention-to-treat patients of 69, 54, 54, 50, and 41 for TNBC, HR^+^/HER2^−^ BC, NSCLC, SCLC, and UC, respectively. NR, median OS not reported at this time.

**Figure 5:**
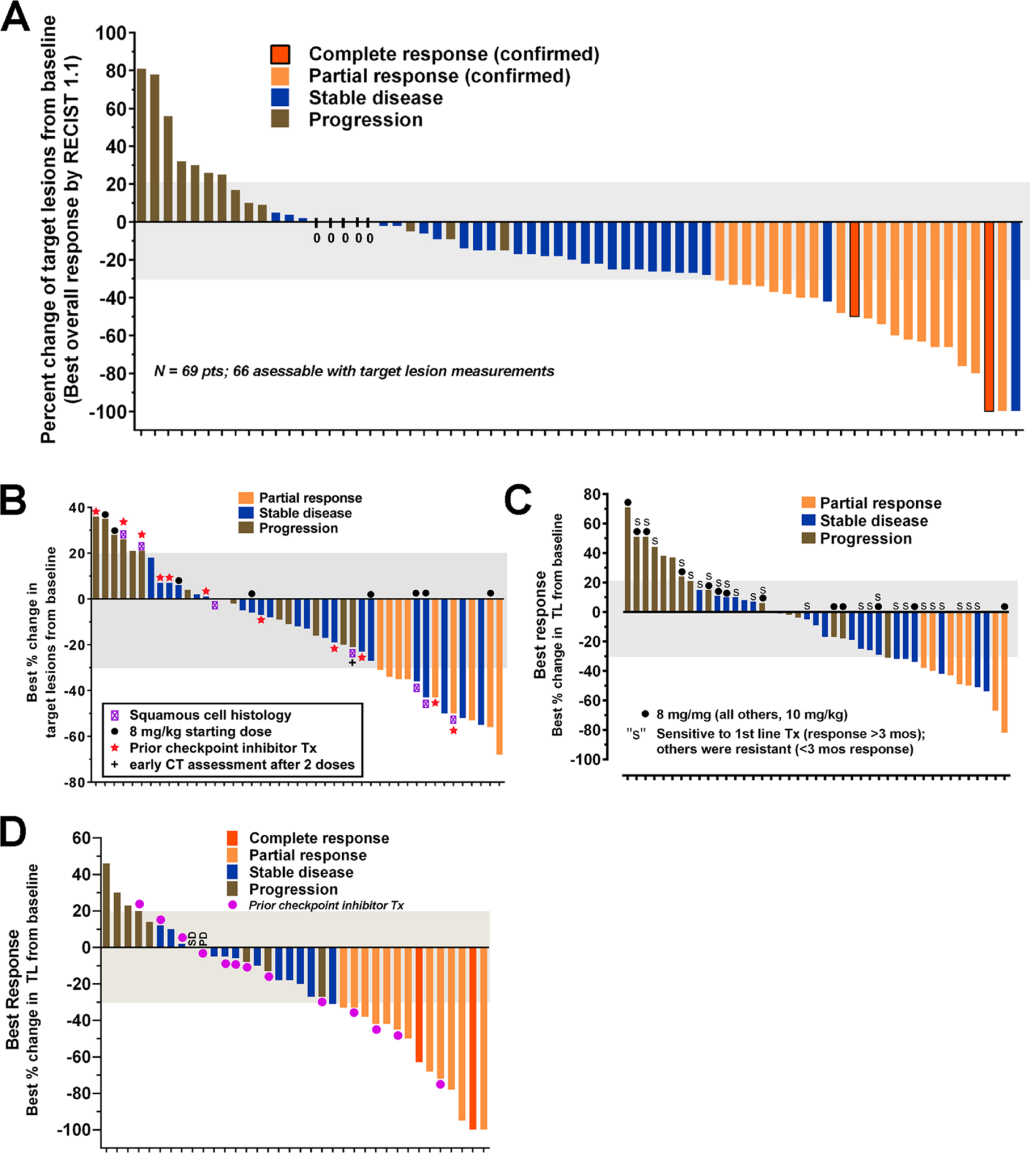
Anti-tumor responses reported in patients with several epithelial cancers who were treated with sacituzumab govitecan, an antibody-drug conjugate targeting TROP-2 Waterfall diagrams depicting the maximum shrinkage observed by investigators in the target lesions selected at baseline after receiving sacituzumab govitecan therapy (response assessment provided only for patients who had at least one follow-up examination). Bar colors provide descriptors for the best overall response achieved in each patient based on RECIST 1.1 criteria. Results are for (**A**) TNBC as adapted from Bardia *et al.* [106], (**B**) NSCLC as adapted from Heist *et al.* [104] (**C**), SCLC as adapted from Gray *et al.* [103], and (**D**) UC as adapted from Tagawa *et al.* [105]. Identification of subpopulations of interest for NSCLC (squamous cell) and SCLC (sensitive vs. resistant to first-line platinum therapy) are provided.

TNBC is a subgroup (~15%) of breast cancer that is not be amenable to hormonal-based therapeutics available for the larger group of breast cancers that express estrogen and/or progesterone, or for those that have elevated human epidermal growth factor-2-(HER2) production [119]. Bardia *et al*. [106] reported the initial therapy results in 69 patients with metastatic TNBC who had received a median of 5 prior therapies. Twenty-one patients (30%) achieved a confirmed objective response (2 complete responses, 19 partial responses), with the median duration of response at the time being 8.9 months (95% confidence interval (CI), 6.1 to 11.3 months). Based on these data, the FDA awarded sacituzumab govitecan breakthrough therapy designation for this indication. Bardia *et al*. [107] updated this experience, expanded to 110 patients, all having had ≥3 lines of prior therapy in the metastatic setting. The confirmed on-site objective response rate was maintained, achieving 34% (3 CR + 34 PR) (Figure 5A), with confirmation by a blinded, independent review of the data achieving a 31% objective response rate. The duration of response was 7.6 months (95% CI, 4.8 to 11.3 months), with 12 of the responders still actively receiving treatment at the time of the analysis. Another important observation was that the duration on the last standard therapy was ≥6 months in only 22/110 (20%) patients, while sacituzumab govitecan was ≥6 months in 41 (37%) patients. This is very encouraging, since it is usual that response rates decrease with subsequent therapies [120].

Initial recent results in a study of 54 advanced patients with heavily-pretreated hormone-positive, HER2-negative, metastatic breast cancer have disclosed an encouraging 31% objective response rate [135].

Assessable patients (*N* = 54) with metastatic NSCLC who received a median of 3 prior therapies had an objective response rate of 19% (Figure 5B), a median duration of response of 6 months (95% CI, 4.8 to 8.3 months), and a clinical benefit rate of 43% that included patients with stable disease for ≥4 months [104]. Eighteen of the patients enrolled received prior checkpoint inhibitor therapy, and for those patients, 2 had a partial response (14%) and 5 had durable stable disease lasting more than 4 months.

Patients with the more aggressive metastatic SCLC, all of whom failed prior platinum/etoposide therapy, showed promising therapeutic responses to sacituzumab govitecan [103]. In this group that included 50 assessable patients receiving 8 or 10 mg/kg, 14% had a partial response (17% for those given the 10 mg/kg dose group) (Figure 5C), with a median duration of response of 5.7 months (95% CI, 3.6 to 19.9 months). Importantly, 20 patients had stable disease with a median duration of 5.6 months (95% CI, 5.2 to 9.7 months). There was a suggested improvement in PR, clinical benefit rate, and progression-free survival in second-line patients who were sensitive to first-line platinum therapy. Further, there was a statistically significant higher overall survival in a subgroup of patients who received prior topotecan therapy *vs.* no topotecan therapy.

Encouraging results also were reported in patients with previously treated stage IV metastatic UC [102, 105], where 14/41 patients (34%; including 2 CR) reported an objective response to sacituzumab govitecan treatment (Figure 5D). Seven of the 14 patients achieving an objective response were continuing treatment at the time of the report, with the duration of response > 6 months in eleven patients, and a duration > 1 year in 6 patients. Fourteen of the enrolled patients had prior checkpoint inhibitor therapy, and of these, 4 (29%) achieved an objective response. In eleven of these patients, sacituzumab govitecan was given as the fourth or later therapy.

In these trials, neutropenia, fatigue, diarrhea, and anemia were the common adverse events, with grade >3 neutropenia being similar to IRI at about 34%. However, grade >3 diarrhea was lower (~9%) than typically found with IRI therapy.

Summarizing, this Trop-2 ADC appears to have good activity as a monotherapy in several cancer types expressing TROP-2, and thus represents a paradigm-change in the ADC technology by: (i) using a moderately-toxic drug, SN-38; (ii) conjugation of the drug to the antibody at a high ratio (~8:1) without affecting targeting and pharmacokinetics; (iii) use of a pH-sensitive, cleavable linker; (iv) administering repeated, high doses of ADC over prolonged times without provoking an immune response; and (v) reduced and manageable toxicities related only to the drug, such as neutropenia and diarrhea.

### PF-06664178; also known as RN927C

Unlike sacituzumab govitecan, RN927C is an ADC composed of a different humanized anti-TROP-2 antibody conjugated to a more potent auristatin derivative that is a microtubule inhibitor at a maximum substitution of 2.0 [108]. In rats that do not express human TROP-2, the toxicity of the conjugate was primarily hematological, but in monkeys that are cross-reactive with human TROP-2, reversible toxicity was observed in multiple epithelial tissues, including the skin, upper alimentary track, and vagina.

A phase I clinical trial with this ADC was performed in 31 patients with a variety of metastatic epithelial cancers [109]. Nineteen of the patients provided tissue samples for TROP-2 expression, with 13 (68%) having medium to high expression (i.e., ≥50% of tissue cells having 2+ to 3+ staining). Treatment was given once every 21 days, starting at 0.15 mg/kg and escalating to 4.8 mg/kg, which was considered intolerable, with 3 patients developing a grade 3 rash, one having grade 4 toxic epidermal necrolysis, and another with grade 3 bullous dermatitis. A lower dose level of 2.4 mg/kg was considered tolerable, but because this dose was considered to lack sufficient therapeutic activity, the trial was stopped before determining the MTD. This ADC was associated with several adverse events, including fatigue, constipation, nausea, chills, infusion-related reactions, neutropenia, rash, weight decrease, arthralgia, decreased appetite, diarrhea, dyspnea, mucosal inflammation, and pruritus, with neutropenia and rash being the most common ≥ grade 3 events. Stable disease was the best response in patients given tolerable doses. Pharmacokinetic analysis revealed the conjugate was stable in serum, but 26/30 patients had post-treatment anti-drug antibody responses.

The safety and efficacy contrast between sacituzumab govitecan and PF-06664178 is quite remarkable and appears to be related to the drug and possibly linkage selection. The highly stable, more potent auristatin-derived ADC appears to have toxicities that are likely associated with TROP-2 targeting, while the less potent and less stable SN-38 conjugate has a safety profile related to its parent drug, IRI, but with an improved therapeutic window [66].

## PERSPECTIVES AND CONCLUSIONS

Although research on TROP-2 dates back almost 40 years [7], there are still many unresolved issues with regard to its role and function in fetal and adult development, as well as in disease, particularly in oncology. Nevertheless, it has already gained an important role in cancer therapy as a predictor of disease activity or prognosis in several cancer types, and indeed appears to have increased expression in a larger number of solid cancers than most other cancer biomarkers. Having extracellular, transmembrane, and intracellular domains implicates TROP-2 in various metabolic pathways, but also permits it to serve as a target for antibody internalization, which is advantageous for delivering cytotoxic agents. Whether TROP-2 expression is critical for antibody targeting and accretion in all neoplasms, how this may be upregulated genetically or epigenetically, and whether it can serve as a target for immunotherapy remain to be studied.

In terms of TROP-2 being a target for immunotherapy, we reported that it is suitable for T-cell therapy using a bispecific antibody that also targets T cells to the tumor [121, 122]. This was anticipated by the demonstration that HLA-restricted cytotoxic T lymphocytes killed antigen-presenting cells expressing TROP-2 [123].

A vaccine to treat murine pancreatic cancer was developed by incorporating TROP-2 into an enveloped virus-like particle [124]. This vaccine resulted in a significant reduction in tumor growth associated with activation of tumor-infiltrating CD4^+^ and CD8^+^ T cells, as well as natural killer cells, in nonclinical studies. When the vaccine was combined with gemcitabine treatment, survival was increased over the respective monotherapies [124].

Our experience with the unconjugated antibody has not shown antitumor activity in non-clinical tumor models, yet both the antibody and sacituzumab govitecan demonstrate antibody-directed cellular toxicity (ADCC) *in vitro* [48, 49, 99, 125–127]. However, a human Fab to TROP-2's extracellular domain has been reported to be active *in vitro* and *in vivo* against MDA-MB-231 human breast cancer cells [57], thus encouraging further studies with other anti-TROP-2 antibodies having different binding epitopes.

Furthermore, our studies to-date have suggested that selecting patients for therapy based on tumor TROP-2 expression is not advantageous, because TROP-2 has a very high (80–90%) expression in most solid epithelial cancers evaluated. However, we have been unable to detect elevated circulating levels of TROP-2 in the patients studied with sacituzumab govitecan, which requires further study possibly with other test formats. Interestingly, using a recombinant cDNA expression library (SEREX) to analyze the serum of Japanese patients with esophageal squamous cancer, 31% of 75 patients showed TROP-2 antibodies [67]. This suggests that TROP-2 is immunogenic in certain patients.

The relapse of cancer patients responding to sacituzumab govitecan, even after continuous therapy for over a year, raises the question of resistance forming either to the drug or to the antibody. This needs to be assessed by evaluating tumor biopsies or circulating tumor cells (CTCs) for expression of TROP-2 or response to SN-38 or IRI during the course of therapy, relating these findings to clinical response. CTCs do express TROP-2, even when EpCAM is diminished or absent [128], suggesting that monitoring of TROP-2 in CTC specimens during therapy may be predictive.

Topoisomerase inhibitors, such as SN-38 and IRI, induce multidrug resistance [129, 130], so it was of interest to explore the use of an ABCG2 inhibitor to overcome this resistance. Chang *et al*. showed that human breast and gastric cancer cell lines made resistant to SN-38 by continuous exposure to this drug *in vitro* could have improved survival *in vivo* when treated with sacituzumab govitecan combined with an ABCG2 inhibitor [131]. Since ABCG2 inhibitors are in clinical development, this may provide another means to improve and prolong efficacy with this ADC.

Tumor resistance also may be overcome or avoided by combining sacituzumab govitecan with other therapeutic agents. The experience with the SN-38 (topoisomerase I) ADC, which causes double-strand DNA breaks by inhibiting homologous recombination repair, motivated us to evaluate combinations with PARP1 (poly [ADP-ribose] polymerase 1) inhibitors, also inducing DNA strand breaks. We found synergism without increased host toxicity in several cases [132]. Certainly, expanding the promising results with sacituzumab govitecan as a monotherapy to combinations with agents currently used in various cancer types, especially in earlier therapy settings, deserves to be studied clinically.

Still another intriguing approach is to induce photothermal ablation of cancer by converting near-infrared laser light to heat. By conjugating hollow gold nanospheres with an anti-TROP-2 antibody that targeted cervical cancer cells (HeLa), the conjugate achieved significant tumor growth-inhibition under laser irradiation. The anti-TROP-2 photothermal therapy was shown to induce apoptosis and DNA damage [133].

Our nonclinical experience measuring TROP-2 expression in human tumor xenografts treated with sacituzumab govitecan suggests that there is a general correlation between quantity of TROP-2 and therapeutic response [113]. This is consistent with the clinical experience [66], which suggests that methods to increase tumor expression of TROP-2 could improve therapeutic efficacy and durability of response if relapsing tumors fail to respond because of downregulation of TROP-2. Using the analysis of circulating tumor cells for TROP-2 expression during therapy, this question could be addressed.

A mesenchymal subset of HNSCC has low TROP-2 and high ErbB3 (HER3) expression, yet an improved anti-cancer activity could be achieved with anti-ErbB3 antibodies [30]. In fact, decreased TROP-2 expression has been associated with HNSCC having sarcomatoid (spindle cell) tumors [52]. These authors also reported that combining antibodies against ErbB-3 and TROP-2 results in synergistic therapeutic responses in human HNSCC xenografts [134].

Summarizing, in addition to TROP-2 serving as a prognosticator for a number of cancer types and its role in controlling cell growth and spread by various signaling pathways, its enhanced expression in many human cancers, with minimal expression in normal tissues, makes it an attractive and novel target for precision medicine approaches, as shown with diverse selective antibody conjugates of radionuclides, toxins, and drugs.

Clinically, the first indication for this ADC is expected to be advanced TNBC [106, 107], but encouraging clinical results have also been reported in NSCLC, SCLC, and UC [102–105], as well as more recently in metastatic hormone-positive metastatic breast cancer [135]. Early and preliminary studies also suggest that TROP-2 may be a suitable target for immunotherapy.

Finally, the recognition that a mutation in *TROP-2* is related to a rare autosomal-recessive hereditary ocular disease, GDLD, has provoked studies to elucidate the mechanism of action resulting in corneal adhesions, which may also disclose the role and function of TROP-2 more generally in various signaling pathways in health and disease. Indeed, the recent implication of TROP-2 in stem cells involved in myocardial infarction and COPD also raise the interest in this molecule beyond cancer. Thus, despite the exact role of TROP-2 in various normal and diseased states still requiring elucidation, the current body of evidence supports its role as a biomarker and selective target in cancer therapy.
